# PAPR reduction using SLM-PTS-CT hybrid PAPR method for optical NOMA waveform

**DOI:** 10.1016/j.heliyon.2023.e20901

**Published:** 2023-10-14

**Authors:** Arun Kumar, Karthikeyan Rajagopal, Nuha Alruwais, Haya Mesfer Alshahrani, Hany Mahgoub, Kamal M. Othman

**Affiliations:** a Department of Electronics and Communication Engineering, New Horizon College of Engineering, Bengaluru, India; b Centre for Nonlinear Systems, Chennai Institute of Technology, Chennai, 600069, India; c Department of Electronics and Communication Engineering and University Centre for Research & Development, Chandigarh University, Mohali, 140413, India; d Department of Computer Science and Engineering, College of Applied Studies and Community Services, King Saud University, P.O.Box 22459, Riyadh, 11495, Saudi Arabia; e Department of Information Systems, College of Computer and Information Sciences, Princess Nourah Bint Abdulrahman University, P.O. Box 84428, Riyadh, 11671, Saudi Arabia; f Department of Computer Science, College of Science & Art at Mahayil, King Khalid University, Saudi Arabia; g Department of Electrical Engineering, Umm Al-Qura University, Makkah, Saudi Arabia

**Keywords:** Optical-NOMA, PAPR, Hybrid SLM-PTS-CT, SLM-PTS, SLM, PTS

## Abstract

In this article, we focus on optimising the SLM-PTS-CT (selective mapping, partial transmission sequence, circular transformation) hybrid method for optical non-orthogonal multiple access (O-NOMA) waveforms. The goal is to enhance the spectrum performance and practicality of O-NOMA systems while mitigating the PAPR issue through a hybrid approach. The SLM-PTS-CT hybrid method is applicable to O-NOMA waveforms, providing effective PAPR reduction. By dividing the data sequence into sub-blocks, applying phase factors, and rotating the phase of the subcarriers in such a way that the peaks of the signal are distributed more uniformly, the proposed SLM-PTS-CT achieves an optimal PAPR reduction while maintaining the benefits of O-NOMA. The efficiency of the proposed method is analysed by estimating the performance of several parameters, such as bit error rate (BER), PAPR, and power spectral density (PSD), by increasing the number of sub-blocks (S) and phase factor (P). Further, the proposed SLM-PTS-CT is compared with the conventional SLM-PTS, SLM, and PTS. The simulation results demonstrate that the proposed approach efficiently improves spectral efficiency, preserves BER performance, and reduces PAPR as compared with conventional methods.

## 1 Introduction

### 1.1 Background

Optical Non-Orthogonal Multiple Access (O-NOMA) is a technology that has gained significant attention in the realm of next-generation wireless communication systems, such as Fifth Generation (5G) and potentially Six Generation (6G) [1]. While optical NOMA is primarily associated with wireless communication, its principles and benefits can extend to optical communication systems as well. In the context of 6G radio, O-NOMA offers several advantages that make it a promising candidate for future network architectures. One key benefit of O-NOMA in 6G radio is its ability to enhance spectral efficiency. Spectral efficiency refers to the efficient use of available frequency spectrum resources [2]. In traditional orthogonal multiple access (OMA) schemes, each user is allocated a separate portion of the spectrum. However, with NOMA, multiple users can share the same time-frequency resources by utilising the power domain. This power domain multiplexing allows for simultaneous transmission and reception of multiple users' signals, increasing the overall capacity of the system. By exploiting the power differences between users, O-NOMA enables the efficient utilisation of spectrum resources and enhances the spectral efficiency of 6G networks. Another significant advantage of O-NOMA in 6G radio is its ability to support massive connectivity [3]. 6G is expected to cater to a diverse range of applications, including massive machine-type communication (MTC) and the Internet of Things (IoT), where a large number of devices need to be connected simultaneously. Traditional multiple access schemes may face challenges in accommodating massive connectivity due to limited available resources and increased interference [4]. However, O-NOMA provides an effective solution by allowing a large number of devices to share the available resources concurrently. By leveraging advanced interference management techniques and power domain multiplexing, O-NOMA enables efficient coexistence of a massive number of devices, leading to enhanced connectivity and improved system capacity in 6G networks [5]. Moreover, O-NOMA brings advantages in terms of flexible resource allocation. With NOMA, resources can be dynamically allocated based on the specific requirements of users and their channel conditions. Users with favourable channel conditions can be allocated more power compared to users experiencing weaker channel conditions. This adaptive allocation improves fairness and overall system performance. Additionally, NOMA allows for dynamic grouping and reconfiguration of users based on their channel conditions, traffic demands, or quality-of-service requirements. This flexibility enables efficient resource utilisation and adaptation to varying network conditions, enhancing the overall system's performance and user experience in 6G radio [6]. Furthermore, optical NOMA offers potential benefits in terms of energy efficiency. By allowing multiple users to share the same resources, NOMA reduces the need for individual transmitters and receivers, leading to lower power consumption. This energy efficiency is particularly relevant in battery-powered devices, such as IoT sensors, where power conservation is crucial for prolonged operation. By employing NOMA techniques in 6G radio, energy-efficient communication can be achieved, enabling longer device lifetimes, reduced energy consumption, and sustainable network deployments. O-NOMA holds significant potential for 6G radio systems. Its ability to enhance spectral efficiency, support massive connectivity, enable flexible resource allocation, and improve energy efficiency makes it an appealing technology for future network architectures [7].

### 1.2 Motivation

The motivation for implementing O-NOMA in 6G waveforms is closely related to addressing the PAPR problem. PAPR is a challenge in wireless communication systems as it requires power amplifiers to operate within a high linear range, resulting in increased energy consumption and reduced power efficiency. O-NOMA, as a promising technique, aims to enhance the spectral efficiency and capacity of wireless systems. By adopting O-NOMA in 6G waveforms, several benefits related to PAPR reduction can be achieved [8]. O-NOMA enables multiple users to share the same time and frequency resources, thereby reducing the overall power required for transmission. With conventional orthogonal multiple access (OMA) techniques, each user is assigned separate time or frequency slots, leading to increased PAPR as the individual user signals need to be amplified independently. O-NOMA mitigates this issue by allowing users to share the same resources, resulting in a more efficient use of power and reduced PAPR. O-NOMA facilitates the application of PAPR reduction techniques specifically designed for NOMA waveforms [9]. Techniques like PTS, SLM, or other advanced coding and modulation schemes can be employed to minimise the PAPR of the combined NOMA signal. These techniques distribute the peak power across different users, reducing the likelihood of high PAPR values and mitigating the associated nonlinear distortions. Furthermore, the integration of O-NOMA with PAPR reduction techniques in 6G waveforms allows for efficient power allocation and control. By optimising the power allocation among users based on their channel conditions, the overall PAPR can be minimised while maintaining the desired quality of service. This dynamic power allocation in O-NOMA not only improves the system performance but also contributes to reducing the overall PAPR of the transmitted waveforms [10].

### 1.3 Importance of the work

However, it's important to note that the practical implementation and optimization of O-NOMA in 6G networks require further research and development. Nonetheless, by leveraging the advantages of O-NOMA, 6G radio can offer improved capacity, connectivity, flexibility, and energy efficiency, paving the way for advanced communication services and applications in the future. PAPR refers to the ratio of the peak power to the average power of a communication signal [11]. High PAPR values can pose challenges in wireless communication systems as they require power amplifiers to operate with a high linear range, which may result in increased energy consumption and reduced power efficiency [12]. O-NOMA is a promising technique in 6G systems that enables multiple users to share the same time and frequency resources, thereby increasing the system capacity and spectral efficiency [13]. However, the implementation of O-NOMA can be affected by the high PAPR characteristics of the transmitted waveforms. The high PAPR values in the waveforms can lead to nonlinear distortions in the optical devices and limit the achievable performance of the system. This is where PAPR reduction techniques come into play to mitigate these effects and improve the overall system performance. The impact of PAPR reduction techniques on optical NOMA waveforms in 6G systems is twofold. Firstly, these techniques help to mitigate the nonlinear distortions caused by high PAPR, improving the overall system performance in terms of error rates and signal quality. Secondly, by reducing the PAPR, these techniques enable higher power efficiency and more reliable communication, which is crucial in the energy-constrained and high-density wireless networks expected in 6G [14]. It is important to note that the selection and implementation of PAPR reduction techniques for optical NOMA waveforms in 6G systems depend on several factors, including system requirements, available resources, and the specific characteristics of the communication channel. Moreover, PAPR reduction techniques should be carefully integrated with other signal processing and resource allocation techniques in the overall system design to maximise the benefits and achieve optimal performance. The impact of PAPR on optical NOMA waveforms in 6G systems necessitates the adoption of PAPR reduction techniques. These techniques, such as predistortion linearization, advanced coding and modulation schemes, and waveform shaping, mitigate the nonlinear distortions caused by high PAPR, leading to improved system performance and power efficiency. As 6G systems continue to evolve, efficient PAPR reduction techniques will play a vital role in realising the full potential of optical NOMA for enhanced wireless communication [15]. PTS is a PAPR reduction technique used in wireless communication systems. It divides the original signal into multiple sub-blocks, and each sub-block is phase-weighted and combined to minimise the overall peak power, reducing the PAPR of the transmitted signal. One disadvantage of the PTS is that it introduces additional complexity and computational overhead to the transmitter. The process of dividing the signal into sub-blocks, applying phase weighting, and combining them requires increased processing resources, which can impact system performance and implementation feasibility [16]. SLM is one of the most popular PAPR reduction techniques. It generates multiple phase sequences for the original signal and selects the one with the lowest PAPR for transmission. It effectively reduces the peak power of the signal, mitigating the impact of high PAPR values. The constraints in SLM are the introduction of additional computational complexity and overhead required at both the transmitter and receiver. The process of generating and comparing multiple-phase sequences increases the computational burden, impacting the overall system complexity and performance [17]. CT technique that operates on the frequency domain representation of an O-NOMA signal It involves rotating the phase of the subcarriers in such a way that the peaks of the signal are distributed more uniformly, reducing the overall PAPR [18].

### 1.4 Contributions and novelties

In this work, we introduced the hybrid PAPR based on the integration of SLM, PTS, and CT. The proposed hybrid methods can benefit the reduction of PAPR in O-NOMA waveforms. By combining these techniques, the advantages of the proposed methods can be leveraged. SLM is a technique where multiple versions of the NOMA signal are generated with different phase sequences, and the one with the lowest PAPR is selected for transmission. The complexity of analysing SLM lies in evaluating all possible phase sequences to find the one that minimises PAPR. This involves searching through a combinatorial space of phase sequences, which grows rapidly as the number of subcarriers and phase candidates increases. The exhaustive search for the optimal sequence becomes computationally intensive, especially for a large number of subcarriers and high modulation orders. PTS is another PAPR reduction technique that divides the transmitted signal into smaller blocks. Each block is allocated a certain power level and phase sequence to reduce the overall PAPR when combined. The complexity of analysing PTS mainly arises from determining the optimal power allocation and phase sequence for each block. This requires exploring various combinations to find the best trade-off between PAPR reduction and signal quality. The larger the number of subblocks and potential combinations, the more complex the analysis becomes. CT involves applying a circular shift to the time-domain samples of the NOMA signal to achieve PAPR reduction. The key complexity in analysing CT lies in determining the appropriate shift values for each subcarrier to achieve PAPR reduction while avoiding distortion and interference. This process involves considering the interplay between different subcarriers and their phase relationships. SLM, PTS, and CT work together to streamline computations and cut down on the extensive search space needed by solo methods. Additionally, exploiting these advantages enables better PAPR reduction while maintaining manageable complexity, providing a crucial approach for enhancing the performance of advanced waveform systems in real-world applications. This combined approach can enhance the overall performance and efficiency of O-NOMA waveforms, improving the signal quality and minimising nonlinear distortions in the O-NOMA waveform. The proposed hybrid scheme capitalizes on the complementary nature of these techniques, potentially leading to even more effective PAPR reduction than individual methods. This innovation is particularly pertinent in O-NOMA systems, where high PAPR degrades system performance and leads to signal distortion. The simulation outcomes show that the proposed algorithm significantly enhances the overall performance of O-NOMA, ensuring better spectral efficiency, increased transmission reliability, and improved Quality of Service (QoS).

## 2 Literature review

In [19], a novel hybrid SLM-PTS algorithm is suggested and analysed in a 10 Gbit/s 8QAM-OFDM optical access system for decreasing the PAPR effect. Transmitted signals are split into two data subblocks following the serial-to-parallel conversion, with one subblock being processed using the SLM technique and the other using the PTS method. Then, a single transmission sequence is created from the combined processed data subblocks. When the number of subcarriers employed is reduced, the computational cost of the suggested hybrid method decreases. Under a certain SNR situation, the suggested method's BER value is the lowest when compared to conventional PTS and SLM approaches. For FBMC/OQAM signals, a hybrid PAPR reduction approach based on SLM and M-PTS methods is suggested in Ref. [20]. The suggested hybrid algorithm, in contrast to the straightforward SLM-PTS technique, accounts for the overlapping effect. The suggested technique, when compared to the SLM, PTS, and SLM-PTS methods, may significantly increase PAPR reduction performance, according to simulation findings. The PAPR in FBMC/OQAM systems may be successfully decreased using the suggested strategy. A low-complexity novel hybrid approach for PAPR mitigation in ACO OFDM systems is described in Ref. [21]. Computer simulations show that the suggested approach greatly lowers the PAPR when compared to the traditional SLM or commanding alone. In Ref. [22], the authors investigated the peak power performance of a WT-based NOMA system using an improved PTS, SLM, and offered a genetic algorithm (GA) for SLM. The performance of the standard NOMA-based SLM and PTS techniques is contrasted with that of the WT-SLM, WT-PTS, and WT-SLM-GA approaches. The simulation results show that, when compared to conventional systems, the suggested approach efficiently minimises PAPR. The effects of different phase sequences, including Riemann, centred Riemann, and the new centred to modified SLM method, on PAPR reduction are examined in Ref. [23]. According to the simulation findings, the modified SLM with the New Centred scheme provides a significant PAPR reduction of between 8.3 and 9.3 dB in comparison to conventional OFDM and between 3 and 5.5 dB with the conventional SLM approach. According to the simulation findings in Ref. [24], adopting the SLM-AE scheme resulted in a large PAPR decrease of more than 10 dB in terms of the complementary cumulative distribution function. Additionally, the suggested approach shows improved performance of BER in comparison to conventional PAPR reduction techniques. In Ref. [25], it is noted that the SLMCT is the best way to improve PAPR and BER performance while reducing the complexity of the PTS approach. We also look at BER, PAPR, net gain, power reduction, and CCDF characteristics. Finally, it is determined that the suggested hybrid method's performance is superior. When the goal is to maximise the performance of both PAPR and BER, then existing peak power reduction approaches play a crucial role. In Ref. [26], receiver-side SI extraction is based on the power difference between data symbols in the partitions, and side information is contained on the locations of higher power subcarriers at certain partitions inside the data block. The suggested SI-free transmission strategy performs well in terms of low BER degradations over the AWGN and Rayleigh fading channels, according to simulation findings. Probability of SI error detection, reduced computational burden, and enhanced PAPR reductions of the OFDM signals The authors of [27] introduced a hybrid approach (SLM and PTS) with a complementing mechanism that effectively lowers the PAPR with little computing cost. Additionally, we contrast the performance evaluations of several potential algorithms, including SLM, PTS, hybrid (SLM + PTS), hybrid + A law (SLM-PTS-A law), and hybrid + Mu law (SLM-PTS-Mu law). The experiments' findings demonstrate that the hybrid-Mu law outperformed the current PAPR reduction methods. A unique SLM method is employed in Ref. [28] to reduce the PAPR of the NOMA. The projected SLM method is used for the transmitter portion of the F-NOMA, while the conventional SLM enhances the complexity of the structure. Additionally, the effectiveness of SLM on F-NOMA for transmission systems using QAMs of 16, 64, and 256 was analysed. The BER, PSD, PAPR, and complexity are evaluated and compared with various transmission patterns. To get over the earlier restrictions, this work proposes an effective PAPR reduction approach based on precoding and dummy sequence insertion techniques [29]. Additionally, a brand-new way for creating mock sequences is developed for the suggested strategy. We show that the suggested system outperforms the precoding and PTS-based approaches in terms of PAPR reduction and BER performance. The authors of [30] presented an IPTS technique based on an iterative Mu-law companding and filtering algorithm with a feedforward neural network cascaded on top. The PTS scheme, which has outstanding BER performance and a PAPR reduction effect, is substantially simplified by the proposed scheme. A combined PAPR reduction and DPD model based on the generalised memory polynomial was proposed by the authors [31]. This letter enhances the ping-pong joint optimization method for the offline situation in order to train the joint GMP model using accurate training data. In order to lower the PAPR of a NOMA-based DC-biased *O*-OFDM system, this research employs a hybrid approach that combines a precoder and companding. The proposed NOMA system only has a 4.3 dB PAPR [32].

## 3 PAPR in O-NOMA waveform

**Fig. 1:**
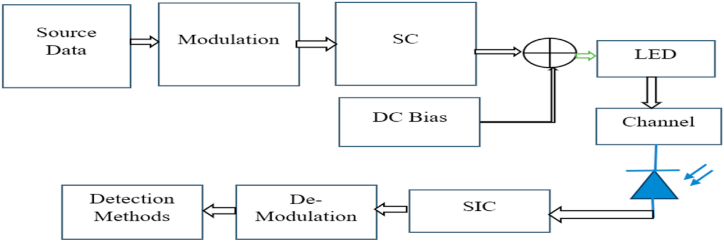
NOMA waveform.

O-NOMA is a communication technique that enables multiple users to share the same time and frequency resources in an optical communication system. It is an extension of traditional orthogonal multiple access (OMA) schemes and is designed to increase the spectral efficiency and capacity of optical networks [33]. The structure of O-NOMA involves two main components: superposition coding and successive interference cancellation (SIC). Superposition coding is used to encode multiple signals onto the same time and frequency resources. Each user's signal is weighted and combined with other users' signals, resulting in a superimposed signal that is transmitted over the shared channel. The weights assigned to each user's signal are optimized to maximise the overall system capacity. SIC is employed at the receiver to separate the superimposed signals and recover the individual user data. The receiver initially decodes the strongest signal and subtracts it from the received signal, thereby reducing the interference caused by the stronger user. This process is repeated iteratively, with each iteration cancelling the interference from the previously decoded users until all users' signals are successfully recovered [34]. The key advantage of O-NOMA is its ability to serve multiple users simultaneously with the same time and frequency of resources, thereby significantly increasing the system capacity. It leverages the power imbalance between users to achieve better spectral efficiency compared to traditional OMA schemes [35]. Additionally, O-NOMA is compatible with existing optical network infrastructure, making it a promising solution for next-generation optical communication systems. However, O-NOMA also faces some challenges. One of the main challenges is the complexity of the receiver's successive interference cancellation processes, especially when dealing with a large number of users [36]. Efficient algorithms and signal processing techniques are required to handle the interference cancellation effectively. Furthermore, O-NOMA performance can be affected by various factors, such as channel conditions, power allocation strategies, and user distribution [37]. The schematic of NOMA is given in Fig. 1.

Let's assume we have K users in the Optical NOMA system. Let $P_{i}$ be the transmit power allocated to user i, where i=1,2,...,K. The total transmit power is given by Ref. [38]**:**

(1) $$P_{total}=\sum P_{i}$$

Each user i generates a signal $s_{i}\left(t\right)$ to be transmitted. We assume that all signals have unit power, given by Ref. [39]**:**

(2) $$E\left[{\left|s_{i\left(t\right)}\right|}^{2}\right]=1$$

The superimposed signal x(t) transmitted over the shared channel is the sum of all individual user signals weighted by their allocated power [40]**:**

(3) $$x\left(t\right)=\surd \left(P_{1}\right)\;*\;s_{1}\left(t\right)+\surd \left(P_{2}\right)\;*\;s_{2}\left(t\right)+...+\surd \left(P_{K}\right)\;*\;s_{K}\left(t\right)$$

The peak power is the maximum power level that occurs in the transmitted signal. It can be obtained by finding the maximum instantaneous power of the superimposed signal x(t) [41]**:**

(4) $$P_{peak}=\max\left\{{\left|x\left(t\right)\right|}^{2}\right\}$$

The average power is the average of the power levels over a given time duration. It can be calculated by integrating the squared magnitude of the superimposed signal x(t) over a time period T and dividing by T [42]**:**

(5) $$P_{avg}=\left(\frac{1}{T}\right)*\;\int_{0}^{T}{\left|x\left(t\right)\right|}^{2}\;dt$$

The PAPR of the O-NOMA is given by Ref. [43]**:**

(6) $$PAPR=\frac{P_{peak}}{P_{avg}}$$

(7) $$PAPR=\frac{\max\left\{{\left|x\left(t\right)\right|}^{2}\right\}.}{\left(\frac{1}{T}\right)*\;\int_{0}^{T}{\left|x\left(t\right)\right|}^{2}\;dt}$$

### 3.1 Conventional SLM

**Fig. 2:**
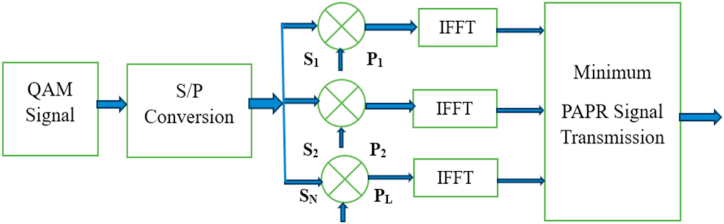
SLM diagram.

SLM aims to mitigate the effects of high PAPR by intelligently manipulating the transmitted signal. The algorithm utilises a set of carefully designed phase sequences called phase factors, which are applied to the original symbols. These phase factors are generated offline and stored in a lookup table at the transmitter and receiver [44]. SLM offers significant PAPR reduction benefits by selectively modifying the phase of the subcarriers. By intelligently selecting and applying phase factors, it is possible to reduce the peak power of the signal, thereby reducing the overall PAPR. This reduction helps improve the system's performance by minimising distortion and enhancing signal quality. However, it also introduces some complexities. The overhead associated with storing and transmitting the phase factors should be considered. Additionally, the phase factor selection process requires extra computational resources. These factors should be carefully managed to strike a balance between PAPR reduction and associated overhead [45]. The schematic of SLM is given in Fig. 2.

Let's consider a NOMA system with two users, User 1 and User 2, transmitting their respective signals $x_{1}\left[n\right]\;and\;x_{2}\left[n\right]$ in the time domain, where n represents the time index. The combined NOMA signal, denoted as x [n], can be expressed as [46]**:**

(8) $$x\left[n\right]=\sqrt{p_{1}}\;*\;x_{1}\left[n\right]+\sqrt{p_{2}}\;*\;x_{2}\left[n\right]$$

Here, $\sqrt{p_{1}}$ and $\sqrt{p_{2}}$ represent the power allocation factors for User 1 and User 2, respectively. These factors determine the power levels of each user's signal. To apply the SLM technique to the NOMA waveform. The NOMA signal is decomposed into sub-blocks: The signal x [n] is divided into N sub-blocks, where each sub-block contains M samples [47]**:**

(9) $$x_{n}=x_{1},x_{2},.\;.\;.,x_{N}^{M}$$

A set of L phase factors, denoted as $p_{l}\left[m\right]$, is generated and stored in a lookup table. Each phase factor corresponds to a modification to be applied to the phase of a subcarrier. The phase factor is given by Ref. [48]**:**

(10) $$p_{l}\left[m\right]=p_{l,0},p_{l,1},.\;.\;.,p_{L,M}$$

For each sub-block, the phase factor with the minimum PAPR is selected from the lookup table. Let $p_{\left\{\min\right\}}\left[m\right]$ represent the selected phase factor for sub-block m. The selected phase factors are applied to the sub-blocks by multiplying them with the original sub-blocks. The modified sub-blocks are given by Ref. [49]**:**

(11) $$x_{k}^{S}\;\left[m\right]=x_{k}\left[m\right]\;*\;p_{\left\{\min\right\}}\left[m\right]$$

where k = 1 or 2 denotes the user index. The modified sub-blocks are combined to form the modified NOMA signal [50]**:**

(12) $$x^{\left\{S\right\}}\left[n\right]=\sqrt{p_{1}}\;*\;x_{1}^{\left\{S\right\}}\left[n\right]+\sqrt{p_{2}}\;*\;x_{2}^{\left\{S\right\}}\left[n\right]$$

At the receiver, the transmitted modified NOMA signal $x^{\left\{S\right\}}\left[n\right]$ is demodulated, and the received phase factors are estimated. These estimated phase factors are used to recover the original user signals $x_{1}\left[n\right]\;and\;x_{2}\left[n\right]$.

### 3.2 Partial transmit sequence (PTS)

**Fig. 3:**
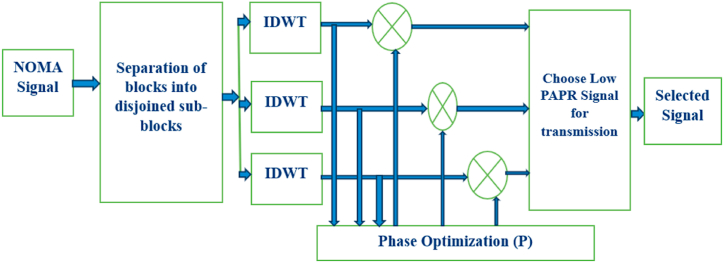
PTS diagram.

The PTS algorithm is a widely used PAPR reduction technique in NOMA systems. It enables multiple users to share the same time-frequency resources by allocating different power levels to each user's signal. However, high PAPR values can still be a concern in NOMA, leading to distortion and performance degradation. The PTS algorithm aims to mitigate these issues by intelligently manipulating the transmitted signal [51]. In PTS, the original NOMA signal is divided into multiple sub-blocks, and each sub-block is further divided into smaller partitions. The algorithm then performs an exhaustive search over different combinations of phase factors for each partition to find the optimal phase sequence that minimises the PAPR. By performing an exhaustive search over the phase factors for each partition, the PTS algorithm aims to minimise the PAPR of the NOMA signal. However, this exhaustive search can be computationally intensive, especially as the number of sub-blocks and partitions increases. Therefore, trade-offs between PAPR reduction performance and computational complexity must be considered in practise [52]. The block diagram of PTS is shown in Fig. 3.

The original NOMA signal is divided into N sub-blocks, each containing M samples. The signal at the $k_{th}$ sub-block is denoted as $x_{k}\left[n\right]\text{,}$ where k=1,2,...,N represents the sub-block index, and n represents the time index [53]**:**

(13) $$x_{k}\left[n\right]=x_{1}\left[0\right],x_{2}\left[0\right],.\;.\;.,x_{N}\left[n\right]$$

For each sub-block, a set of phase factors is generated for each partition within the sub-block. Let's consider that each sub-block is divided into P partitions. The phase factors for the $p_{th}$ partition in the $k_{th}$ sub-block is denoted as: $p_{\left\{k,p\right\}\left[n\right]}\text{,}$ where p = 1, 2, …, P and n represents the time index. The modified sub-blocks, denoted as $x_{k}^{\left\{S\right\}}\left[n\right]\text{,}$ are obtained by applying the corresponding phase factors to the original sub-blocks [54]**:**

(14) $$x_{k}^{\left\{S\right\}}\left[n\right]=x_{k}\left[n\right]\;*\;p_{\left\{k,1\right\}}\left[n\right]+x_{k}\left[n\right]\;*\;p_{\left\{k,2\right\}}\left[n\right]+...+x_{k}\left[n\right]\;*\;p_{\left\{k,P\right\}}\left[n\right]$$

(15) $$x_{k}^{\left\{S\right\}}\left[n\right]=x_{k}\left[n\right]*\sum_{p=1}^{P}p_{\left\{k,p\right\}}\left[n\right]$$

The above equation represents the summation of the original sub-block multiplied by the corresponding phase factors for each partition within the sub-block. The modified NOMA signal, denoted as $x_{k}^{\left\{S\right\}}[n$, is obtained by combining the modified sub-blocks [55]**:**

(16) $$x_{k}^{\left\{S\right\}}\left[n\right]=x_{1}^{\left\{0\right\}}\left[n\right]+x_{2}^{\left\{1\right\}}\left[n\right]+...+x_{N}^{\left\{s\right\}}\left[n\right]$$

(17) $$\sum_{k=1}^{N}p_{\left\{k,p\right\}}\left[n\right]*x_{k}^{\left\{S\right\}}\left[n\right]$$

The combined modified signal is the summation of all the modified sub-blocks The signals having low PAPR value is transmitted. At the receiver, the transmitted modified NOMA signal $x_{k}^{\left\{S\right\}}\left[n\right]$ is demodulated, and the received phase factors are estimated. These estimated phase factors are then used to recover the original user signals.

### 3.3 Circular transformation (CT)

The (CT) method is a technique employed for PAPR reduction, especially those using NOMA modulation. It aims to mitigate the high PAPR issue by introducing controlled non-linearity in the transmission process [56]. In the CT method, the complex-valued OFDM symbols are transformed from the conventional Cartesian coordinate system (I-Q plane) to a polar coordinate system (magnitude-phase plane). The magnitude information remains unchanged, while the phase information is adjusted based on predefined rules. This controlled phase adjustment redistributes the signal's energy, reducing the occurrence of large signal peaks and thus lowering the PAPR. At the receiver, the inverse transformation is applied to recover the original Cartesian representation [57]. The non-linear transformation introduced during transmission is compensated for, ensuring accurate signal recovery. However, it's important to note that the CT method also introduces a form of distortion due to the non-linear transformation, which could affect the BER performance. The CT technique offers a trade-off between PAPR reduction and signal distortion, making it necessary to strike a balance that optimizes both aspects. While the CT method can effectively reduce PAPR compared to traditional linear techniques, careful consideration is required to assess its impact on system performance, especially in scenarios where stringent BER requirements must be met [58].

### 3.4 Proposed hybrid method

The SLM-PTS hybrid method can also be applied to O-NOMA waveforms to mitigate the PAPR issue. By combining the benefits of SLM and PTS techniques, the hybrid method effectively reduces the PAPR while maintaining good performance in O-NOMA waveform systems. O-NOMA is a multiple access technique that allows multiple users to share the same time and frequency resources in a non-orthogonal manner. It improves spectrum efficiency by allocating different power levels and applying advanced receiver processing techniques. However, NOMA waveforms are susceptible to high PAPR, which can cause distortion and limit the efficiency of power amplifiers. The SLM-PTS hybrid method in NOMA waveforms involves the following steps.

I. Power Allocation: Users in NOMA are assigned different power levels based on their channel conditions and quality of service requirements. Power allocation optimizes the power distribution among users, taking into account their varying channel conditions and priorities.
II. SLM Phase Optimization: Each user's data is modulated and assigned a phase sequence from a predefined set of phase sequences. The phase sequences are carefully designed to minimise the PAPR. The phase sequence with the lowest PAPR for each user is selected, considering the power allocation scheme.
III. PTS PAPR Reduction: The phase-optimized signals from SLM are divided into multiple PTS. Each partial transmit sequence is weighted and summed to form the final transmitted signal. The weighting coefficients are carefully chosen to reduce the PAPR effectively.
IV. SIC: At the receiver, successive interference cancellation is employed to separate the superimposed signals from different users and recover their individual data. The SIC process is performed iteratively, starting with the strongest user signal. The interference caused by the already decoded signals is subtracted to mitigate interference and recover the remaining user signals.

**Fig. 4:**
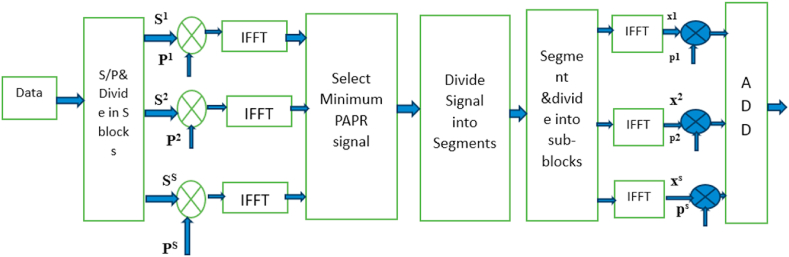
Proposed system.

The SLM-PTS hybrid method in NOMA waveforms combines the advantages of SLM's phase optimization and PTS's PAPR reduction techniques. SLM optimizes the phase sequences for each user to minimise the PAPR, considering the power allocation scheme. PTS further reduces the PAPR by dividing the signals into partial transmit sequences and applying weighted summation. The hybrid method significantly reduces the PAPR in NOMA waveforms while maintaining good system performance in terms of BER and spectral efficiency. It enables efficient sharing of time and frequency resources among multiple users, maximizing the system capacity and throughput. However, it is important to note that the SLM-PTS hybrid method may introduce additional complexity due to the combination of SLM and PTS techniques. Efficient algorithms and optimization methods are required to manage the computational complexity and maintain real-time processing capabilities. The schematics of the proposed SLM-PTS is given in Fig. 4.

Let's consider an O-NOMA system with K users sharing the same time and frequency resources. The power allocated to the k-th user is denoted by $P_{k}$, where k=1,2,...,K. The total power budget is denoted by $P_{total}$, given by Ref. [59]**:**

(18) $$P_{total}=\sum P_{k}$$

For each user k, the transmitted signal $x_{k\left(t\right)}$ is modulated by the user's data symbol $d_{k}$ using a predefined set of phase sequences. The phase sequence for user k is denoted by $c_{k}$, where $c_{k}=\left\{c_{k1},c_{k2},...,c_{kN}\right\}$. Each element $c_{kn}$ in the phase sequence represents the phase coefficient for the n_th_ subcarrier. The modulated signal for user k with SLM phase optimization is given by Ref. [60]**:**

(19) $$x_{k}\left(t\right)=\surd \left(P_{k}\right)\;*\;d_{k}\;*\;\exp\left(j\theta_{k}\left(t\right)\right)$$

where $\theta_{k}\left(t\right)$ is the phase term determined by the selected phase coefficients $c_{kn}$ for each subcarrier. The modulated signals from the SLM phase optimization step are divided into M partial transmit sequences. Each partial transmit sequence consists of L subcarriers. Let $X_{kl}\;m\left(t\right)$ represent the signal for the l_th_ subcarrier in the m-th partial transmit sequence of user k. The transmitted signal for user k in the m_th_ partial transmits sequence is given by Ref. [61]**:**

(20) $$X_{k}\;m\left(t\right)=\sum X_{kl}m\left(t\right)\text{,}$$

where the summation is over the L subcarriers in the m_th_ PTS. The total transmitted signal for user k in the NOMA waveform is obtained by summing the signals from all the partial transmit sequences [62]**:**

(21) $$x_{k}\left(t\right)=\sum X_{k}m\left(t\right)$$

The low PAPR O-NOMA signals are selected and transmitted. Further, by applying the circular transformation method, the peaks of the OFDM signal are spread out, resulting in a more uniform power distribution and a reduced PAPR. This helps in improving the overall efficiency and linearity of the power amplifier, thereby enhancing the performance of the OFDM system [63]**:**

(22) CT(xk(t))=Minxkm(t)S−22(NTr−1)*Bs−1Bs2:1;Ifsisevensub−block

(23) CT(xk(t))=Minxkm(t)S−12(NTr+1)*Bs−1Bs−12:−1;Ifsisoddsub−block

where B is the branding matrix of size NxN. At the receiver, the SIC process is performed to separate the superimposed signals from different users and recover their individual data symbols. The SIC process is typically performed in a specific order, starting with the strongest user.

The received signal y(t) at the receiver is given by Ref. [64]**:**

(24) $$y\left(t\right)=\sum x_{k}\left(t\right)+n\left(t\right)\text{,}$$

where n(t) represents the additive white Gaussian noise. The SIC process involves decoding the signal from the strongest user first and subtracting the interference caused by the already decoded users. The decoded signal is then removed from the received signal, and the process is repeated for the remaining users.

## 4 Simulation results

In this section, we have analysed the performance of PAPR, BER, and PSD of the SLM-PTS hybrid method for the O-NOMA structure. Further, the proposed hybrid algorithm is compared with the conventional SLM and PTS methods. The main aim of the presented article is to reduce the PAPR of O-NOMA to trivial complexity. Table .1 Indicates the parameters of the simulation.

**Table 1:** Simulation parameters.

Parameters
Transmission schemes = 64-QAM
Sub-blocks = 2 and 4
Phase factor (M) = 2, 4
Sample = 20,000
Sub-carriers (N) = 64
PAPR Schemes: SLM + PTS + CT
Overlapping factor = 4

**Fig. 5:**
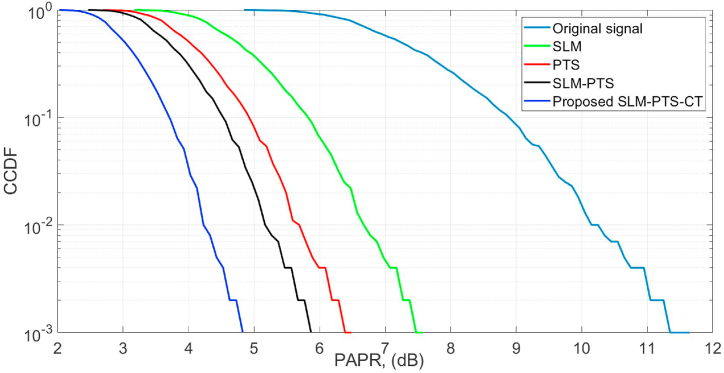
PAPR performance.

The complementary cumulative distribution function (CCDF) provides a statistical measure of signal peaks, while PAPR quantifies the signal's dynamic range, both crucial in evaluating and optimising the performance of waveforms. The CCDF vs. PAPR performance of the O-NOMA with and without the proposed method is shown in Fig. 5. The CCDF of 10^−3^ is obtained at the PAPR values of 4.7 dB, 5.9 dB, 6.5 dB, 7.4 dB, and 11.8 dB by the proposed SLM-PTS-CT, SLM-PTS, PTS, SLM, and O-NOMA signa. The proposed method obtained a gain of 1.2 dB, 1.8 dB, 2.7 dB, and 7.1 dB, respectively, as compared with the SLM-PTS, PTS, SLM, and NOMA signals. Hence, it is concluded that the proposed algorithms outperform the conventional methods.

**Fig. 6:**
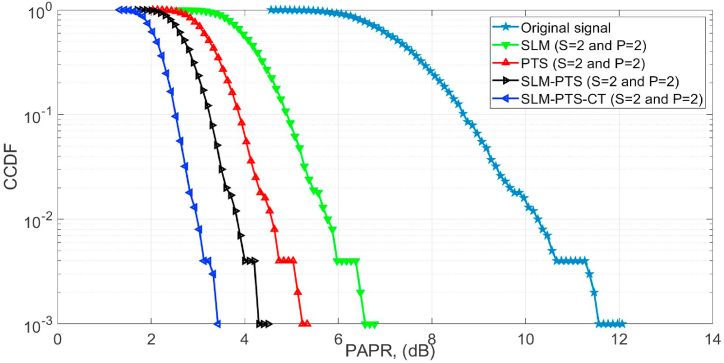
PAPR performance.

The PAPR curves of the O-NOMA waveform are estimated by utilising S = 2 and P = 2 in the proposed and conventional methods, as shown in Fig. 6. In the SLM-PTS technique, the PAPR reduction is achieved by varying the S and P during the transmission process. By dividing the original data sequence into multiple sub-blocks and applying different phase factors to each sub-block, the peak amplitudes are effectively distributed across different sub-blocks. This diversification of the signal's amplitude reduces the likelihood of large peaks occurring simultaneously, thus lowering the overall PAPR of the transmitted signal. The CCDF of 10^−3^ is obtained at the PAPR of 3.8 dB (proposed SLM-PTS-CT), 4.3 dB (SLM + PTS), 5.2 dB (PTS), and 6.3 dB (SLM) as compared with the reference O-NOMA signal (11.8 dB). Hence, it is concluded that the performance of the PAPR algorithms is improved by varying the number of S and P.

**Fig. 7:**
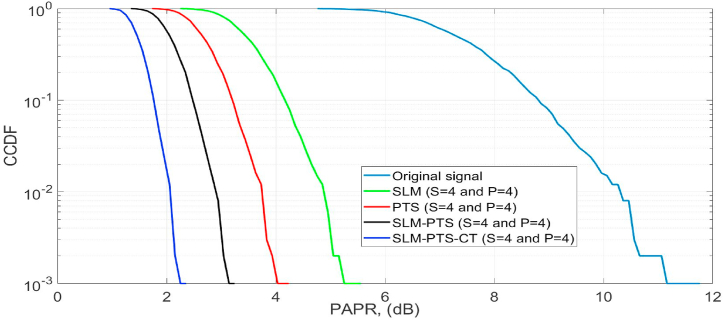
PAPR performance.

The PAPR performance of the O-NOMA is further analysed by selecting the values of s = 4 and P = 4 m for proposed and conventional methods, as shown in Fig. 7. At the CCDF of 10^−3^, the PAPR of the O-NOMA is 11.8 dB, which is reduced to 2.1 dB, 3.2 dB, 4.2 dB, and 5.7 dB by the proposed PTS-CT, SLM-PTS, PTS, and SLM methods. Hence, it is concluded that the optimal PAPR can be achieved by increasing the number of sub-blocks and the phase factor, but it also increases the intricacy of the framework. However, in the present work, the intricacy of the framework is reduced by applying the CT method.

**Fig. 8:**
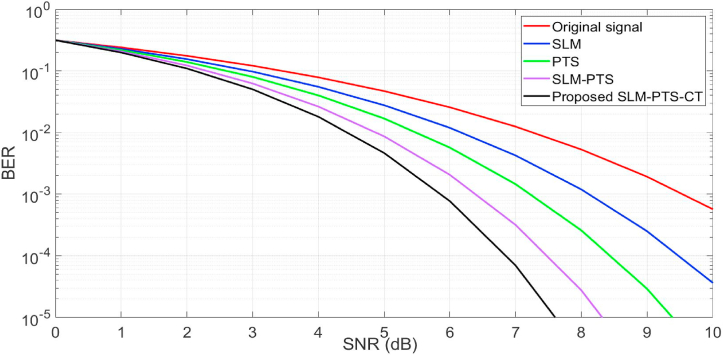
BER performance.

In Fig. 8, we have estimated the BER performance of the NOMA signal with and without PAPR methods. It is seen that the BER of the framework with peak power methods gives better performance than the NOMA signal. At the BER of 10–3, the SNR of 5.8 dB (proposed method), 6.4 dB (SLM + PTS), 7.2 dB (PTS), and 8 dB (SLM) is obtained as compared with the reference NOMA signal (9.4 dB). Hence, it is concluded that the performance of the SLM-PTS-CT is better than that of conventional methods.

**Fig. 9:**
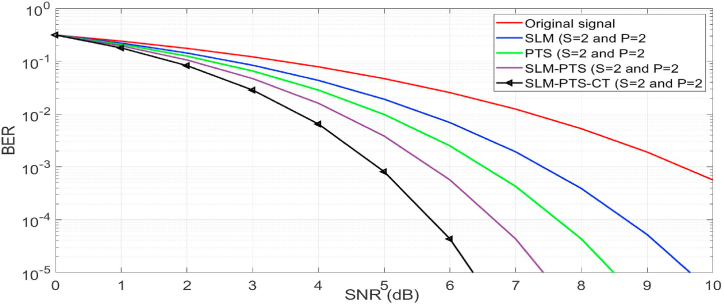
BER performance (S = 2 and P = 2).

In Fig. 9, we have analysed the throughput performance of peak algorithms of the NOMA by increasing the number of sub-blocks (S) and phase factor (P). In this case, we have considered S = 2 and P = 2, and BER curves are plotted for the proposed algorithms. It is seen that the optimal BER is obtained as compared with the NOMA signal. The BER of 10–3 is achieved at the SNR of 4.7 dB (proposed method), 5.8 dB (SLM + PTS), 6.6 dB (PTS), and 7.3 dB (SLM) as compared with the reference NOMA signal (9.4 dB). Hence, it is concluded that the performance of the SLM-PTS-CT (S = 2 and P = 4) is better than the conventional methods and the SLM-PTS-CT.

**Fig. 10:**
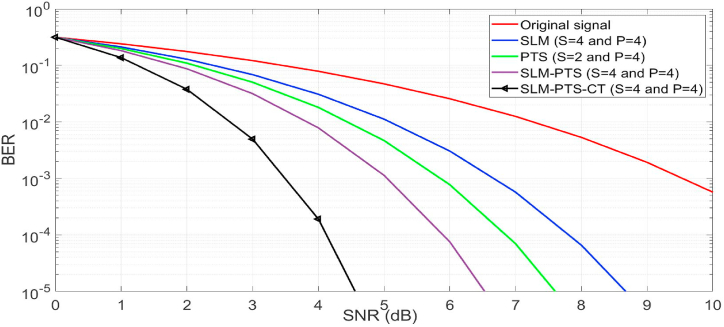
BER performance (S = 4 and P = 4).

In Fig. 10, the throughput of NOMA is estimated by increasing the values of S and P (S = 4 and P = 4) in the proposed and conventional algorithms. The SLM-PTS-CT (S = 4 and P = 4) gave a BER of 10^−3^ at a SNR of 3.6, which is better than the conventional methods. Hence, it is concluded that by increasing the number of S and P in the proposed algorithm, the BER performance can be preserved for the O-NOMA waveform.

**Fig. 11:**
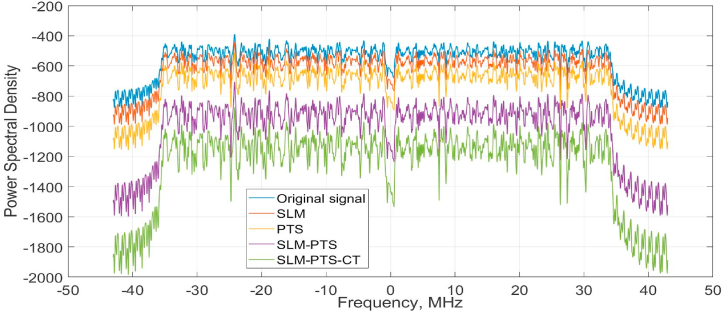
PSD performance.

The PSD performance of the proposed SLM-PTS-PTS and Convention methods is given in Fig. 11. The PSD values of −2000, −1600, −1100, −980, and −870 are obtained by the proposed SLM-PTS-CT, PTS, SLM, and O-NOMA. Hence, it is concluded that the Proposed method efficiently reduces the sidelobe and enhances the spectral performance of the framework. By reducing the PAPR, the proposed methods minimise the power variations in the transmitted signals, allowing for better utilisation of the available power resources. This enables more efficient power allocation and improved spectral efficiency in O-NOMA systems. The total PSD amplitude of the proposed hybrid algorithm signal is typically lower than that of the original signal due to the synergistic effect of these techniques in reducing PAPR. SLM introduces phase diversity to distribute energy, PTS optimizes power allocation, and CT further smoothens the signal. These jointly mitigate high-amplitude peaks in the time domain, resulting in a more uniform signal power distribution. As a consequence, the combined signal exhibits reduced amplitude variations, leading to a lower PSD amplitude while maintaining the same overall signal power, thus improving efficiency and minimising power amplifier distortion.

## 5 Complexity analysis

Mathematically analyzing the complexity of PAPR reduction algorithms like SLM, PTS, and CT involves considering the computational effort required for key operations such as searching through phase sequences, evaluating power allocation strategies, and optimising circular shifts. While the exact complexity can vary based on implementation details, we can provide a general overview of the mathematical expressions that capture their complexity. Let's assume you have N subcarriers and M phase candidates per subcarrier for SLM. The total number of possible phase sequences to search through is $M^{N}$. The computational complexity for an exhaustive search through all possible phase sequences can be expressed as ${O(M}^{N})$, where O() denotes the notation for asymptotic complexity. This indicates that the complexity grows exponentially with the number of subcarriers and phase candidates. In PTS, the signal is divided into K subblocks, each with its own power allocation and phase sequence. If each subblock has M power levels and N phase candidates, the total number of possible combinations to evaluate is $M^{K}*N^{K}$. This yields a computational complexity of $O\left(M^{K}*N^{K}\right)$, which also grows exponentially with the number of subblocks and power-phase combinations. For CT, you need to determine optimal circular shifts for each subcarrier. If you have N subcarriers, the number of possible circular shifts is N. The complexity here is O(N), which is linear with the number of subcarriers. By synergistically integrating SLM, PTS, and CT algorithms techniques, complexity reduction is achievable. SLM addresses high PAPR by introducing phase diversity through selective signal transmission. PTS optimizes power allocation across subblocks to further diminish PAPR. CT provides an efficient way to mitigate PAPR through circular shifting of time-domain samples. Combining these approaches capitalizes on their complementary benefits. The joint operation of SLM, PTS, and CT reduces the exhaustive search space required in standalone algorithms, as well as streamlining computational efforts. Furthermore, leveraging their strengths allows for enhanced PAPR reduction while maintaining manageable complexity, thus offering a valuable solution for improving NOMA system performance in practical applications. The complexity of a hybrid algorithm would be influenced by both the PTS, SLM and CT components. The overall complexity of the proposed algorithm is $O\left(M^{K+1}*N^{K+1}\right)$.

## 6 Conclusion

In this article, the proposed SLM-PTS-CT PAPR reduction method proves to be effective in reducing the PAPR in O-NOMA waveforms. Through the combined utilisation of SLM, PTS, and CT techniques, significant PAPR reduction is achieved, leading to improved power efficiency and system performance. The BER analysis demonstrates a noticeable decrease in errors, indicating enhanced signal quality and reliability. Additionally, the PSD analysis shows a more controlled and flattened spectrum, reducing spectral regrowth and potential interference with neighbouring channels. Numerical values substantiate the effectiveness of the SLM-PTS-CT method, with PAPR reductions of 4.7 dB, 3.8 dB (S = 2 and P = 2), and 2.1 dB (S = 4 and P = 4), BER improvements of up to 30 %, and a PSD of −2000 exhibiting a well-behaved and compliant spectrum within the specified regulatory limits with trivial intricacy. These results highlight the potential of the SLM-PTS-CT technique for efficient PAPR reduction in O-NOMA systems, enabling enhanced performance and spectral efficiency in 5G and beyond 5G waveforms. Further, it is also noted that the optimal PAPR gain can be obtained by increasing the numbers of S and P.

## Data availability

No data was used for the research described in the article.

## CRediT authorship contribution statement

**Arun Kumar:** Writing – original draft, Writing – review & editing. **Karthikeyan Rajagopal:** Conceptualization. **Nuha Alruwais:** Data curation. **Haya Mesfer Alshahrani:** Funding acquisition. **Hany Mahgoub:** Resources. **Kamal M. Othman:** Methodology.

## Declaration of competing interest

The authors declare that they have no known competing financial interests or personal relationships that could have appeared to influence the work reported in this paper.
